# Preventive measures of obstetric Fistula: knowledge and practice among service providers in two Nigerian Health Institutions

**DOI:** 10.4314/ahs.v24i4.22

**Published:** 2024-12

**Authors:** Chikaodili Ndidiamaka Ihudiebube-Splendor, Nonyelum Nnenna Jisieike-Onuigbo, Paulina Chigwara Chikeme, Onyinyechi Lilian Utazi, Anulika Jennifer Nnamani

**Affiliations:** 1 Department of Nursing Sciences, Faculty of Health Sciences and Technology, University of Nigeria Nsukka Enugu Campus, Nigeria; 2 Department of Internal Medicine, College of Health Sciences and Technology, Nnamdi Azikiwe University Awka Nnewi Campus, Nigeria

**Keywords:** Knowledge, obstetric fistula, practice and prevention

## Abstract

**Background:**

Obstetric fistula remains a major public health problem which serves as a proxy indicator of the status of Nigerian women and of the availability and accessibility to quality maternal health services. This study aims to assess the knowledge, practice and perceived factors that might hinder preventive interventions to reduce obstetric fistula by service providers in Abakaliki, Ebonyi State Nigeria.

**Methods:**

A cross-sectional survey of 169 service providers (doctors (49) and nurses (120)) in selected health facilities in Abakaliki was conducted using a validated self-administered questionnaire.

**Results:**

Majority (71%) of the participants were registered nurse/midwife while only 29% were doctors. Most (87.0% & 89.9%) of the participants had both adequate knowledge and good practice of preventive measures of obstetric fistula respectively. Major factors perceived to hider the practice of obstetric fistula preventive measures were patient-related factors (mean=2.97) and institutional factors (mean = 2.51). There was significant association between age (p = 0.008), marital status (p = 0.029), profession (p = 0.039), years of experience of work (p = 0.003) and the knowledge of obstetric fistula while none of the demographic characteristics had association (p > 0.05) with the practice of obstetric fistula preventive measures.

**Conclusion:**

Although most participants in this study had adequate knowledge and good practice of obstetric fistula preventive measures, there is still a great need to train and retrain doctors and nurses on current guidelines for obstetric fistula prevention and conservative management. Government should also strengthen the health facilities at all levels to provide emergency obstetric and newborn care.

## Introduction

Obstetric fistula (OF) is a serious reproductive health challenge for women in the developing world.^1^ It is a traumatic childbirth injury that occurs when labor is obstructed and delivery is delayed.^2^ Globally, it is conservatively estimated that more than two million young women live with untreated obstetric fistula, while between 50,000 and 100,000 new cases are affected each year.^3^ The prevalence of obstetric fistula in West Africa ranges from 1-4 per 1,000 deliveries and the annual fistula incidence is estimated to be 2-11 per 1000 birth. Nigeria contributes to 40% of the global burden of the disease 4,5, and earlier reports showed that about 400,000 to 800,000 women are living with obstetric fistula and about 20,000 new cases of obstetric fistula occur every year.^3^ A hospital prevalence rate of 0.44 per 100,000 deliveries was also reported from a Teaching Hospital in Abakaliki, Southeast Nigeria.^6^

A prolonged obstructed labor is the single most causal factor for obstetric fistula. There are other factors such as female genital mutilation, violent rape, early child bearing, adolescent pregnancy and surgeries, amongst others^6^. A prolonged obstructed labour leads to the destruction of the tissues that normally separate the bladder or the rectum from the vagina and creates a passageway (fistula) through which either urine or faeces leaks continuously^1^,^7^. The true burden of fistula may remain difficult to determine especially in poor resource settings such as Africa primarily because of the associated stigma. Victims of obstetric fistulae are usually the lucky survivors of traumatic prolonged childbirth which often times results in fetal loss. They become social outcasts; some are divorced and rejected by their families. They travel long distances in search of treatment, which often eludes them. Some have to take to begging or prostitution for survival.^4^

While the condition has disappeared in developed and industrialized countries where emergency obstetric care and universal high-quality maternity care are widely available, it remains a source of concern in resource-poor nations as tens of thousands of new fistula sufferers are added to the millions of pre-existing cases each year.^2^ Although obstetric fistula are treatable and preventable, its eradication in Nigeria presents an enormous challenge, partly because of the financial resources required and more so because of shortages of appropriately trained personnel and the uneven distribution of Nigeria's skilled health workforce. Other important factors include: non-availability and accessibility of appropriate emergency obstetric care during labour and delivery, lack of specialist hospitals, non-training of health care workers, and insufficient equipment.^4^,^8^

The recent accelerated fistula repair campaigns and outreach services by the Federal Ministry of Health, development partners and nongovernmental actors has raised treatment of women with OF to approximately 6,000 repairs annually in Nigeria.^4^ At this rate, it will take many years and huge investments to clear the current backlog of women suffering from this devastating condition. This challenge underscores the importance of preventing fistula from occurring in the first place. The complexity of this problem in Nigeria and the multi-factorial determinants of the condition calls therefore, for a more multi-disciplinary and multi-sectoral approach to tackle the social concerns and address the status of Nigerian women's rights and the availability and access to quality maternal health services.

The Federal Ministry of Health in Nigeria has developed a Standard of Practice (SOP) for the management of obstetric fistula for doctors and nurses. Trainings, surveys and needs assessments were conducted to inform prevention by the insertion of an in-dwelling catheter (for 4 to 6 weeks) to relieve pressure on the bladder following prolonged obstructed labour. It is estimated that between 40% and 95% of small fresh fistulae heal spontaneously with Foley's catheter insertion for 4 to 6 weeks 1,4,9. Obstetric fistulae can also be repaired surgically unless the fistulae are too large or there is associated damage to other tissues which makes repair impossible. 4,10

However, despite government's efforts in establishing a fistulae repair center in Ebonyi State and training of doctors and nurses, obstetric fistula scenario has remained a persistent scourge in Ebonyi State. Hence, the knowledge of healthcare providers on OF prevention is crucial to planning advocacy for identification of potential risk factors, as well as ensuring prompt medical intervention especially emergency obstetric services (EmOC) when needed. This helps to promote positive behavioral values that limit the burden of OF, and its associated social and psychological challenges in the community.^11^ Knowing and understanding various obstetric fistula management strategies by doctors and nurses are the main key to prevention of its occurrence among women of child bearing age especially to those who are pregnant and in labour. Much as this sounds relevant, there are dearths of publication on the knowledge and practice of health workers in preventing this health challenge in this part of the country.

The study was therefore aimed to assess the knowledge and practice of preventive measures of obstetric fistula among doctors and nurses that render maternal health services in selected health facilities in Abakaliki, Ebonyi State, Nigeria and also to identify factors that might hinder doctors' and nurses' practice of these preventive measures.

## Methods

### Study Design

This is a cross-sectional survey involving one hundred and sixty nine (169) service providers doctors (49) and nurses (120) working in the Obstetrics and Gynaecology Unit of Federal Teaching Hospital, (FETHA) and National Obstetric Fistula Centre (NOFIC), Abakalikii from 1^st^ March to 30th April 2018. Data was collected using researchers-developed questionnaire which comprised 40 items on demographic characteristics of participants, knowledge about OF, preventive measures utilized,and perceived factors that hinder the practice of preventive measures of OF. The instrument was validated by three experts from Departments of Nursing Sciences and Measurement and Evaluation, University of Nigeria Nsukka. The instrument was pilot-tested among eighteen (18) doctors and nurses from VVF Center Calabar. The data obtained from pilot testing were analyzed using Cronbach's alpha reliability test which yielded a reliability coefficient of 0.81. Ethical clearance was obtained from Research and Ethics Committee of National Obstetric Fistula Center Abakaliki and written informed consent was obtained from the participants. The collected data were coded, categorized and entered into Microsoft Excel Windows 7 and exported to IBM SPSS (Statistical Package for Social Sciences) Version 20.0 (Chicago 11 USA) software for analysis. Data were subjected to simple descriptive statistics of frequency, percentages, mean and standard deviations. Test of association of demographic variables were done using Chi-square test. Probability value less than 0.05 was considered statistically significant. The results were presented in tables.

In this study, knowledge of OF was defined as the ability of the participants to define OF, types, identify the causes and predisposing factors associated with it and was measured at three-point levels: Adequate (knowledge score 6 – 7 items); Moderate (knowledge score 4 – 5items) and Inadequate (knowledge score 0 – 3items). Practice of preventive measures in this study was defined as actions taken to limit or eradicate the occurrence of OF in terms of monitoring labour using partograph, use of indwelling catheter, counseling on birth spacing, childhood and women nutrition, birth preparedness and family planning among others. Practice was measured at three-point levels: Good practice (practice score 8 – 10items); Moderate practice (practice score 5 – 7items) and Bad practice (practice score 0 – 4items).

## Results

### Socio-demographic characteristics of the participants

One hundred and sixty-nine (169) participants successfully filled and returned the questionnaire. The mean (standard deviation) age of participants was 32.3 (7.6) years. More than half 112 (66.3%) were females and 108(63.9%) were married. Majority 90(71.0%) were registered nurse/midwife, 21(12.4%) were house officers, 24(14.2%) were registrars, and 4(2.4%) were consultants. Based on the number of years of work experience, 56(33.1%) had 6 – 10 years' work experience, while 19(11.2%) had more than 20 years' work experience. The minimum education attained was diploma 83(49.1%); 59(34.9%) had their first degree, 21(12.4%) possessed Masters Degree while 6(3.6%) had Ph.D. (Table 1).

**Table 1:** Socio-demographic Characteristics of study participants (n=169)

Variables	Frequency	Percentage (%)
**Age (Years)**		
23 – 27	39	23.1
28 – 32	61	36.1
33 – 37	32	18.9
38 – 42	26	15.4
43 – 47	11	6.5
**Mean(SD) = 32.3(7.6)**		

**Sex**		
Male	57	33.7
Female	112	66.3

**Marital status**		
Single	58	34.3
Married	108	63.9
Divorced	0	0.0
Widowed	3	1.8

**Profession**		
Registered Nurse/Midwife	120	71.0
House Officer	21	12.4
Registrar	24	14.2
Consultant	4	2.4

**Years of work experience**		
0 – 5 years	38	22.5
6 – 10 years	56	33.1
11 – 15 years	31	18.4
16 – 20 years	25	14.8
More than 20 years	19	11.2

**Educational attainment**		
Diploma	83	49.1
Degree	59	34.9
Masters	21	12.4
Ph.D	6	3.6

### Knowledge about obstetric fistula among Doctors and Nurses

Table 2 showed that all 169(100%) the participants had heard about obstetric fistula. Most 149(88.1%) of the participants stated their training school/hospital was their source of information about obstetric fistula. All 169(100%) the participants correctly defined obstetric fistula as an opening between the bladder and/or rectum and the vagina; Most 147(87.0%) participants affirmed that VVF is the most common type of obstetric fistula.

**Table 2:** Knowledge about obstetric fistula among Doctors and Nurses (n=169)

Items	Doctors	Nurses	Frequency	Percentage (%)
**†Have you heard about obstetric fistula?**				
Yes	49	120	169	100.0
No	0	0	0	0.0
**If yes, what is your source of information?**				
Radio	7	9	16	9.5
Television	2	2	4	2.4
Newspaper	0	0	0	0.0
Training school/Hospital	40	109	149	88.1
**†What is obstetric fistula?**				
An opening between the bladder and vagina only	0	0	0	0.0
An opening between the rectum and vagina only	0	0	0	0.0
An opening between the bladder and/or rectum and vagina	49	120	169	100.0
**†What is the common type of obstetric fistula?**				
Vesico-vaginal fistula (VVF)	37	110	147	87.0
Recto-vaginal fistula (RVF)	12	10	22	13.0
†What are the underlying factors associated with occurrence of obstetric fistula? *				
Poverty	22	26	48	10.9
Female genital mutilation/cutting	49	93	142	32.1
Lack of education	13	19	32	7.2
Early marriage/childbirth	42	49	91	20.6
Poor nutrition	6	5	11	2.5
Lack of access to health care	43	75	118	26.7
†What do you think is the most common cause of obstetric fistula? *				
Prolonged obstructed labour	49	118	167	52.2
Caesarean section	0	0	0	0.0
Forceps delivery	43	96	139	43.4
Diseases and Radiotherapy	7	7	14	4.4
†Tick the three delays associated with the occurrence of obstetric fistula? *				
Delay in deciding to seek care	49	120	169	33.3
Delay in reaching the health care facility	49	120	169	33.3
Delay in receiving adequate care	49	120	169	33.3
**†What is the age group mostly affected with obstetric fistula?**				
Less than 15years	49	120	169	100.0
15 – 24 years	0	0	0	0.0
More than 24 years	0	0	0	0.0
**Overall Knowledge**				
Adequate *(knowledge score 6 – 7 items)*			147	87.0
Moderate*(knowledge score 4 – 5items)*			22	13.0
Inadequate *(knowledge score 0 – 3items)*			0	0.0

* **= Responses are not mutually exclusive**

† **Variables used to assess obstetric fistula knowledge**

The common underlying factors associated with the occurrence of obstetric fistula identified by majority of the participants were: female genital mutilation/cutting {142(32.1%)}, lack of access to health care {118(26.7%)} and early marriage/childbirth {91(20.6%)}. The most common causes of obstetric fistula by the participants included: prolonged obstructed labour {167(52.2%)} and forceps delivery {139(43.4%)}. All 169(100%) the participants stated the three delays associated with the occurrence of obstetric fistula as: delay in deciding to seek care 169(33.3%), delay in reaching the health facility 169(33.3%) and delay in receiving adequate care 169(33.3%). All 169(100%) affirmed that the age group mostly affected with obstetric fistula are those less than 15 years.

The result of the overall level knowledge showed that most 147(87.0%) of the participants had adequate knowledge on obstetric fistula as they responded correctly to more than 6 items (75%) in the subscale while only 22(13.0%) had moderate knowledge.

### Practice of preventive measures of obstetric fistula by doctors and nurses

Table 3 revealed major preventive measures of obstetric fistula utilized by doctors and nurses to include: easy access to emergency obstetric care 164(97.0%); passing of an indwelling catheter in cases of prolonged and/or obstructed labour 156(92.3%); counseling women on the use of family planning/giving quality antenatal and intranatal care 152(89.9%); skilled professional attendance during delivery 151(89.3%), educating women on birth preparedness and complication readiness 148(87.6%) and identification of danger signs of pregnancy, labour and delivery for prompt referral 147(87.0%). Based on the number of responses to the items in the subscale, majority 152(89.9%) of the participants had good practice of preventive measures of obstetric fistula as they responded correctly to more than 8 items, while 17(10.1%) had moderate practice.

**Table 3:** Practice of Preventive measures of obstetric fistula by doctors and nurses (n=169)

Preventive measures of obstetric fistula utilized	Doctors	Nurses	Total	Percentage
Used partograph to monitor progress of labour	47	50	97	57.4
Passed an indwelling catheter in cases of prolonged/and obstructed labour	41	115	156	92.3
Identification of danger signs of pregnancy, labour and delivery for prompt referral	47	100	147	87.0
Community involvement and advocacy	20	22	42	24.9
Counselling women on use of family planning methods	49	103	152	89.9
Educating women on birth preparedness and complication readiness	48	100	148	87.6
Giving quality antenatal and intranatal care	43	109	152	89.9
Girl child and women nutrition education	31	60	91	53.8
Immediate response to emergency obstetric conditions	49	115	164	97.0
Skilled professional attendance during delivery	47	104	151	89.3
**Overall Practice**				
Good practice *(practice score 8 – 10items)*			152	89.9
Moderate practice *(practice score 5 – 7items)*			17	10.1
Bad practice *(practice score 0 – 4items)*			0	0.0

*Responses are not mutually exclusive

### Factors that hinder the practice of preventive measures of obstetric fistula

Table 4 showed that the major factors that hinder the practice of preventive measures of obstetric fistula among doctors and nurses were patient-related factors and institutional/organizational factors with Mean of means scores of 2.97 and 2.51 respectively.

**Table 4:** Factors that hinder the practice of preventive measures of obstetric fistula (n=169)

Variables	Strongly Agreed	Agreed	Disagreed	Strongly Disagreed	
Patient-related Factor					
Socio-economic status of the patient	67	102	0	0	3.40
Cultural beliefs of patient	32	87	41	9	2.84
Lack of family support (husband)	21	68	55	25	2.51
Poor antenatal visit	32	109	21	7	3.11
Patient's level of education (ignorance and illiteracy)	43	94	21	11	3.00
**Mean of means**					2.97*
**Health workers-related factor**					
Lack of training	9	31	81	48	2.01
Time constraints	14	51	72	32	2.28
Poor staff strength (man power)	29	71	42	27	2.60
**Mean of means**					**2.30**
**Institutional/Organizational-related Factor**					
Distance of health facility	27	75	44	23	2.63
Hospital protocols	39	81	31	18	2.83
Lack of appropriate equipment	11	49	81	28	2.25
Non-availability of emergency obstetric care	38	91	22	18	2.88
Lack of suitable equipped health facility.	9	31	72	57	1.95
**Mean of means**					2.51*

**Decision rule: Mean score ≥ 2.5 is accepted**

* **Accepted Mean of means scores**

### Association between demographic characteristics and level of knowledge of obstetric fistula

The Chi-square test of association in Table 5 revealed that there was significant association between age (p = 0.008), marital status (p = 0.029), profession (p = 0.039), years of experience of work (p = 0.003) and the knowledge of obstetric fistula while sex (p = 0.241) and educational attainment (p = 0.605) had no association with the knowledge of obstetric fistula.

**Table 5:** Association between demographic characteristics and level of knowledge (n=169)

Demographic characteristics	Level of Knowledge		Total	X^2^ (p-value)
	Adequate knowledge	Moderate knowledge		
**Age range**				13.618
23 – 27 years	28	11	39	(0.008) *
28 – 32 years	54	7	61	
33 – 37 years	28	4	32	
38 – 42 years	26	0	26	
43 – 47 years	11	0	11	
**Sex**				1.369
Male	52	5	57	(0.241)
Female	95	17	112	
**Marital status**				7.064
Single	45	13	58	(0.029)*
Married	99	9	108	
Divorced	0	0	-	
Widowed	3	0	3	
**Profession**				10.084
Registered	78	12	90	(0.039)*
Nurse/Midwife				
House Officer	13	8	21	
Junior Registrar	14	1	15	
Senior Registrar	8	1	9	
Consultant	4	0	4	
**Years of work experience**				20.844 (0.003)*
0 – 5 years	25	13	38	
6 – 10 years	51	5	56	
11 – 15 years	28	3	31	
16 – 20 years	24	1	25	
More than 20 years	19	0	19	
**Educational attainment**				1.843
Diploma	75	8	83	(0.605)
Degree	50	9	59	
Masters	17	4	21	
PhD	5	1	6	

* **Significant values**

### Association between demographic characteristics and practice of preventive measures of obstetric fistula

Results in Table 6 showed that there was no significant association between age (p = 0.177), sex (p = 0.885), marital status (p = 0.600), profession (p = 0.296), years of work experience (p = 0.128), and educational attainment (p = 0.392) and the practice of the preventive measures to obstetric fistula.

**Table 6:** Association between demographic characteristics and practice of preventive measures of obstetric fistula (n=169)

Demographic characteristics	Practice of preventive measures		Total	X^2^ (p-value)
	Good practice	Moderate practice		
**Age range**				11.9504
23 – 27 years	29	10	39	(0.177)
28 – 32 years	58	3	61	
33 – 37 years	28	4	32	
38 – 42 years	25	1	26	
43 – 47 years	9	2	11	
**Sex**				0.0208
Male	21	6	57	(0.885)
Female	101	11	112	
**Marital status**				0.6003
				(0.600)
Single	53	5	58	
Married	96	12	108	
Divorced	0	0	-	
Widowed	3	0	3	
**Profession**				4.9114
Registered Nurse/Midwife	80	10	90	(0.296)
House Officer	18	3	21	
Junior Registrar	11	4	15	
Senior Registrar	9	0	9	
Consultant	4	0	4	
**Years of work experience**				7.1477
0 – 5 years	31	7	38	(0.128)
6 – 10 years	49	7	56	
11 – 15 years	30	1	31	
16 – 20 years	23	2	25	
More than 20 years	19	0	19	
**Educational attainment**				2.9968
Diploma	78	5	83	(0.392)
Degree	51	8	59	
Masters	18	3	21	
PhD	5	1	6	

## Discussion

In the past several years, declining quality of maternal health care and rising poverty levels have been implicated in causing a rise in the incidence of obstetric fistula in Nigeria. 11 Obstetric fistula became a rarity in the developed world since the development of standard obstetric care. Prevention of obstetric fistula is dependent on knowledge, participation and uptake of quality services. Keeping this in view, the present study was focused on assessing the knowledge and practice of preventive measures of obstetric fistula and factors that might hinder health workers from practice of such preventive measures in selected hospitals in Abakaliki, Ebonyi State, Nigeria. In the present study, 147 (87.0%) of the participants had adequate knowledge of obstetric fistula and its prevention. The adequate knowledge about obstetric fistula among doctors and nurses cannot be separated from the fact that the participants were health workers whose source of information was basically from their training institutions and experiences in the practice area. This finding was comparable with previous reports from Addis Ababa and Zamfara, Nigeria where 67% and 88% respectively of skilled birth attendants were found to have good knowledge of obstetric fistula and its prevention.^12^,^13^ Good knowledge in these studies was significantly associated with in-service training on obstetric fistula prevention and management, resource availability and number of years of clinical experiences.

The findings of this study also indicated that majority 152 (89.9%) of the participants had good practice of the preventive measures as shown by the use of partograph to monitor the progress of labour in 93.5% of the participants. Other preventive measures practiced by majority of the participants include: passing of an indwelling Foley's catheter for 7 to 14 days or longer for women who had obstructed labour, implementation of birth preparedness and complication readiness plan for a pregnant woman and education of pregnant women during every antenatal visit on the importance of birth preparedness and complication readiness. The good practices reported by the participants might be linked to their awareness and adequate knowledge about obstetric fistula condition. Also the settings being tertiary institutions that predominantly manage obstetric fistula, it is assumed that the medical staff working in these units might have been involved in some training and update programmes on standard of care for obstetric fistula and its prevention. This result is similar to the findings published earlier where 66.2% of skilled birth attendants in Addis Ababa, Ethiopia^14^ and 85.4% in Zamfara State, Nigeria^15^ had good practice of obstetric fistula preventive measures. However, this result disagrees with a report from Zambia where only few (24% and 15%) of their nurses knew that partograph and prolonged catheterization respectively can be used in primary prevention of obstetric fistula 16. It is possible that the facility used for their study may not have been managing fistula cases and therefore needs training in obstetric fistula care.

Findings from this study also revealed that patient and institutional/organizational factors were two major impediments to the practice of obstetric fistula preventive measures by the participants. The patients' factors identified were: poor socio-economic status of the patient, cultural beliefs, lack of family support and poor antenatal visit while the institutional/organizational factors were: poor access to the health facility, hospital protocols, and non-availability of emergency obstetric care services. Studies have shown that social and economic causes that indirectly lead to the development of obstetric fistula are issues of poverty, poor nutrition, lack of education, early marriage and childbirth, harmful traditional practices such as female genital mutilation, and lack of good quality or accessible maternal health care amongst others^17^. Poverty being number one indirect cause hinders women from accessing normal and emergency obstetric care because of long distances and expensive procedures. For some women, the closest maternal care facility can be more than 50km away. The findings in this present study supports the reports from Knartoum, Sudan^18^ that identified socio-economic status of the patient, cultural beliefs, lack of family support, poor antenatal visit, woman's level of education, distance of health facility, hospital protocols, and non-availability of emergency obstetric care as major constraints to obstetric fistula preventive practices. The present study showed that some demographic characteristics of the participants such as age, marital status, profession and years of experience positively affect the knowledge of doctors and nurses towards obstetric fistula and its prevention but do not have any influence on their practice. While comparing participants' education and knowledge of obstetric fistula and practice of its preventive measures, the results appear non-significant which is possibly due to unequal number of doctors and nurses used as participants. Some previous studies have similar results as the present study 12,13.

However, continuous education programmes in the field of standard care practices are recommended for concerned health workers in Obstetric Units while a basic level necessary training for all medical and nursing students must be provided during their training programmes. This will include information about all the currently used obstetric fistula preventive measures. It is hoped that the findings of our study will provide a solid platform for planning strategies for improving women healthcare services, implementing emergency obstetric services in acceptable and planned manner especially in the rural areas, thus reducing preventable deaths associated with obstetric fistula in our communities. More so, emphasis will be on empowering the women educationally and economically, with special consideration for the girl child. Education increases the knowledge of women on preventive measures to take and increases their decision-making skills. Furthermore, government can help strengthen the health system by providing a suitable work environment and appropriate equipment for health workers to work with.

## Limitations of the study

The study was conducted only in the health facilities that admit and manage obstetric fistula cases where majority of the doctors and nurses by virtue of working there, must have gained some experiences or been exposed to training in obstetric fistula management which is not the case for other health workers in other facilities within the geopolitical zone. Hence, the survey results cannot be generalized to represent the entire Nigerian doctors and nurses.

There was also a possibility of response bias, in which the participants might have tended to give socially desirable responses when questioned on their practices/use of obstetric fistula preventive measures. Based on quantitative design of the study, there was no intention to collect and analyze participant's experiences.

## Recommendations

Based on the findings of this study, the following has been recommended-

- Further research is warranted to evaluate health workers implementation of urethral catheterization for prevention and conservative management of obstetric fistula.
- Incorporation of the obstetric fistula preventive and conservative management guideline into the curriculum of training institutions for Nigerian doctors and nurses/midwives.
- Government should strengthen the health facilities at all levels to provide Emergency obstetric and newborn care (EmONC)
- Training/retraining and supportive supervision on use of the partograph, urethral catheterization and EmONC by doctors and nurses/midwives.
- Household and community barriers should be addressed by community sensitization and awareness creation on obstetric fistula prevention, male involvement and women's education and empowerment.

## Conclusion

Although the survey results revealed that majority (87.0% & 89.9%) of the participants had adequate knowledge about obstetric fistula and a good practice of its preventive measures respectively, this study provides valuable evidence that continuous education and update programmes in the field of standard care practices for concerned health workers in Obstetric Units will help in improving women healthcare services, thus reducing preventable death associated with obstetric fistula in rural communities in Nigeria.
